# Jellyfish Venom-Induced Cardiotoxicity and Immune Responses: Mechanisms and Potential Therapeutic Strategies

**DOI:** 10.3390/md23100369

**Published:** 2025-09-23

**Authors:** Yueyue Li, Zhiwen Qiu, Bingbing Li, Xiaoyu Geng, Xuelu Yu, Yue Li, Wei Li, Jishun Yang

**Affiliations:** 1 Naval Medical Center of PLA, Naval Medical University, Shanghai 200052, China; yueyueli921@163.com (Y.L.); m18131452435@163.com (B.L.); 15339625597@163.com (X.G.); selinayxl@163.com (X.Y.); 2 Department of Nanomedicine & Shanghai Key Laboratory of Nautical Medicine and Translation of Drugs and Medical Devices, Naval Medical University, Shanghai 200433, China; qzw0531@126.com; 3 School of Health Science and Engineering, University of Shanghai for Science and Technology, Shanghai 200093, China; 18337018029@163.com

**Keywords:** jellyfish, envenomation, toxin, immunology, cardiotoxicity, therapy

## Abstract

Jellyfish stings represent a significant global marine hazard, causing injuries from localized skin damage to fatal systemic complications. While skin reactions are the most common symptom, heart toxicity (cardiotoxicity) is the primary cause of death. A growing body of evidence shows that the immune system’s response worsens this venom-induced heart damage. However, current research remains disproportionately focused on cutaneous inflammatory responses, leaving systemic immunopathological processes—especially those potentiating cardiotoxicity—poorly understood. Moreover, few jellyfish toxins (like those from the *Chironex fleckeri*) have been thoroughly studied, and the molecular mechanisms of heart injury remain largely unknown. This review introduces a novel pathophysiological classification of jellyfish envenomation into three distinct categories—immunotoxicity-dominant, cardiotoxicity-dominant, and dual-mechanism synergistic—based on clinical and mechanistic profiles. By synthesizing current knowledge on venom components and their multi-system interaction, we aim to identify actionable therapeutic targets and propose mechanism-driven treatment strategies. This refined classification offers a foundation for future clinical decision-making and the development of targeted therapies, potentially improving patient outcomes through more personalized envenomation management.

## 1. Introduction

**Figure 1:**
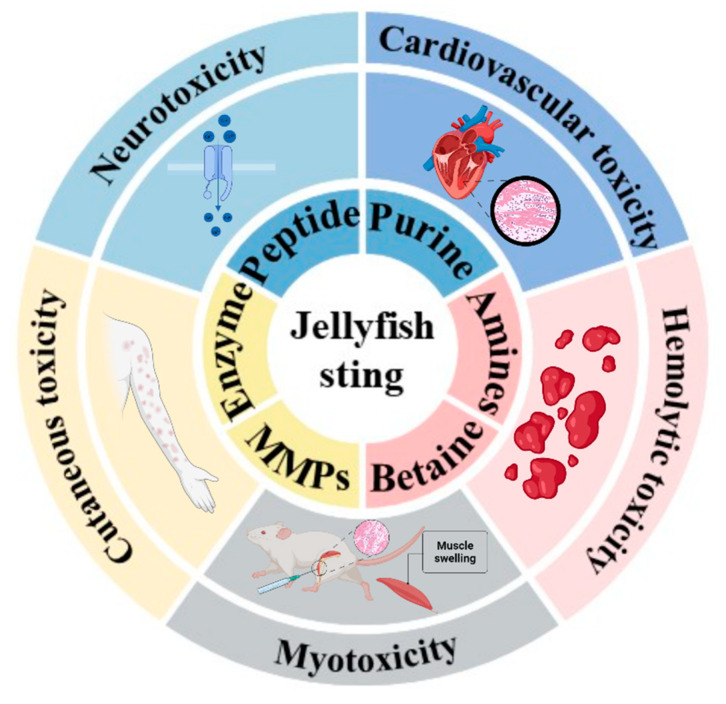
Schematic diagram of major toxic components and pathogenic effects of jellyfish venom. Jellyfish venom contains various proteins—including peptides, enzymes, and matrix metalloproteinases (MMPs)—and non-protein compounds such as purines, biogenic amines, and betaines. These toxins can induce multiple toxic effects, including cardiovascular toxicity, hemolytic toxicity, dermal toxicity, neurotoxicity, and myotoxicity. Created with BioRender.com.

Jellyfish, as ancient members of the phylum Cnidaria, have evolved complex venoms consisting of multiple bioactive toxin components, which mediate diverse pathological effects ranging from localized tissue necrosis to life-threatening systemic envenomation [1,2]. In recent years, jellyfish blooms have become more frequent worldwide due to climate change, eutrophication, and human activities [3], posing significant threats to human health. Against the broad global context of increasing jellyfish blooms, coastal areas—particularly public beaches, seaside resorts, and tourist vacation zones—are increasingly becoming high-risk regions for jellyfish–human encounters and related envenomation incidents [4,5]. According to Boulware, an estimated 150 million jellyfish stings occur worldwide each year [6]. Regionally, in China’s coastal waters alone, *Cyanea nozakii* is responsible for hundreds of thousands of envenomation cases annually [7]. When the skin comes into contact with a jellyfish, individuals typically experience immediate local pain, followed by the development of wheals [8]. Although rare, in some cases, exposure to jellyfish venom can trigger anaphylactic shock [9]. Envenomation can lead not only to irritation [10] but also to severe pain, hemolysis, neurotoxicity, myotoxicity, and cardiac injury (Figure 1). While dermatotoxicity is the most common early symptom [11], cardiac injury is the most serious complication and the primary cause of jellyfish sting-related mortality [12]. Evidence shows that various jellyfish toxins, especially those from cubozoan species, cause both direct and indirect damage to cardiac tissue, leading to myocardial dysfunction and injury [13,14].

Critically, jellyfish venoms exhibit pronounced interspecies variability and possess complex, multi-component compositions. For instance, the venom of *Chironex fleckeri* is dominated by pore-forming toxins such as CfTX-1/2, which cause life-threatening cardiac toxicity in humans [14], whereas the venom of *Nemopilema nomurai* is rich in phospholipases A2 and metalloproteinases that contribute to severe inflammatory reactions and tissue damage [15]. Current research on jellyfish envenomation remains predominantly focused on localized cutaneous inflammatory responses, with most studies exclusively associating venom-induced immune reactions with dermatological manifestations [16,17]. However, the mechanisms underlying jellyfish venom-mediated cardiotoxicity remain poorly understood. Jellyfish envenomation-induced cardiotoxicity may be aggravated by concurrent inflammatory responses and oxidative stress [15,18]. This review systematically evaluates representative jellyfish species, with particular emphasis on venom-triggered immune responses and cardiotoxic effects. Through systematic analysis of toxic venom constituents, we delineate and contrast their distinct immunotoxic and cardiotoxic mechanisms. The synthesized evidence provides valuable therapeutic targets for developing drugs against both venom-induced immune dysregulation and cardiac damage, thereby advancing treatment strategies for jellyfish envenomation.

## 2. Jellyfish Morphology and Nematocyst Ultrastructure

Jellyfish, members of the subphylum Medusozoa within the phylum Cnidaria, exhibit a simple anatomical organization characterized by a mesogleal layer sandwiched between two epithelial tissues (the epidermis and gastrodermis). Their bodies are translucent and flexible, consisting of four main parts: an umbrella-shaped bell, oral arms, tentacles, and accessory organs [19]. Bell diameter varies widely across species, ranging from 2 cm to 2 m. Situated under the bell, the oral arms facilitate both feeding and waste expulsion [20]. Tentacle number and shape also differ significantly among species and are entirely absent in *rhizostomeae* [21]. As an ancient member of this phylum, jellyfish inherently possess cnidocysts. Their tentacles and perioral tissues are densely populated with specialized nematocytes—stinging cells with unique structural and functional characteristics [22]. These cells play pivotal roles in various biological processes, including prey capture, defense mechanisms, locomotion [23], and mating behaviors [24]. Each nematocyte contains a highly specialized organelle called the nematocyst, which serves as both the venom reservoir and delivery apparatus.

**Figure 2:**
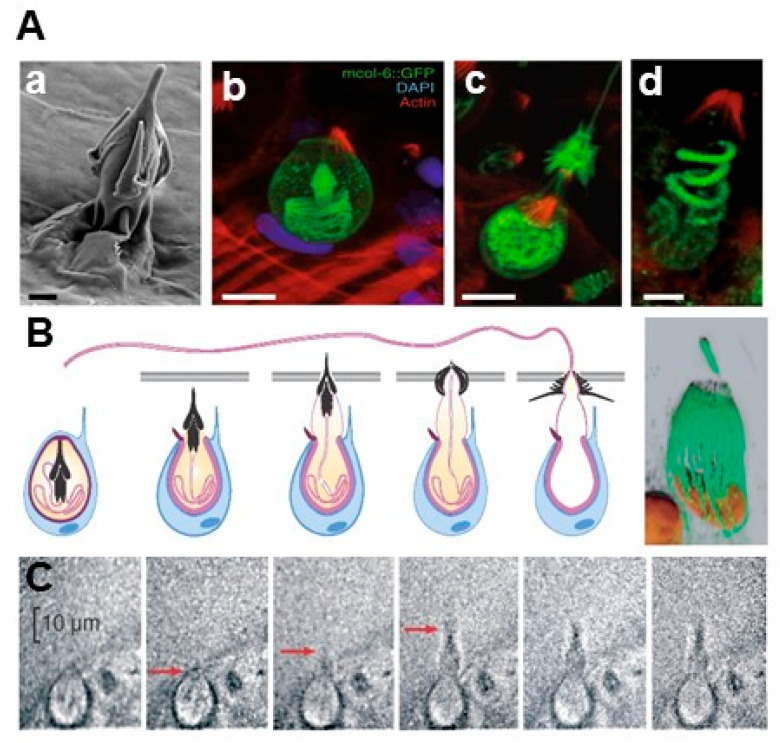
Nematocyst discharge characterization: (**A**) Ultrastructural and minicollagen distribution analyses of *Hydra* nematocytes (stinging cells). Scale bars: 1 µm in (**a**,**d**); 5 µm in (**b**,**c**) [32]. (**B**) Schematic mechanism. Each nematocyte (blue) harbors one cyst (pink) with stylets that punch a hole into prey. (**C**) Framing-mode sequence @1,430,000 fps (Hamamatsu C4187); arrows indicate progress of discharge [33].

The nematocyst is a complex secretory organelle derived from Golgi vesicles [25,26]. It consists of a collagenous capsule enclosing a tightly coiled, rapidly evertible tubule—known as the nematocyst thread—which may be either permeable or impermeable. These stinging organelles are distributed differentially across cnidarians: typically concentrated on tentacles [27], they are found mainly on the oral arms in scyphozoans such as *Pelagia noctiluca*, though they may also occur on the bell and body [28]. Structurally, the nematocyst comprises three key components: the operculum, capsule, and thread. Prior to discharge, the thread remains coiled within the capsule. Upon stimulation, it everts at extreme speeds—completing deployment in approximately 700 nanoseconds, with velocities reaching 18.6 m/s [29] (Figure 2). This phenomenon, termed discharge, represents one of the fastest biological events known [30]. Nematocysts function as dual chemosensory and mechanosensory cells [7,16], responsive to both physical and chemical cues. During discharge, the thread accelerates instantaneously at up to 5.4 × 10^6^ g, producing impact pressures of 7.7 GPa [31]. This allows it to penetrate diverse biological barriers, from human skin to crustacean exoskeletons. Once embedded, the barbed tubule delivers venom efficiently into the target, initiating rapid envenomation for both predation and defense.

## 3. Jellyfish Venom Composition

Jellyfish venom is the primary cause of toxic reactions and lethal outcomes following stings. Recent ecological changes, including global increases in jellyfish blooms and associated sting incidents, have driven significant research efforts to characterize the venom’s bioactive components. Nevertheless, the full composition remains poorly characterized, with only a limited number of constituents clearly identified (Table 1). Biochemically, the venom is a complex mixture of structurally novel and highly potent molecules. It is composed mainly of peptides and proteins, but also contains thermolabile enzymes, vasoactive amines (e.g., histamine and 5-hydroxytryptamine), kinin-like compounds, bioactive lipids, and other low-molecular-weight effectors [34]. Both proteinaceous and non-proteinaceous fractions contribute to the venom’s diverse toxic effects—including cardiac injury.

### 3.1. Matrix Metalloproteinases

Metalloproteases are a class of proteolytic enzymes dependent on metal ions (particularly Zn^2+^ and Ca^2+^), which are widely distributed in the venoms of various animals such as centipedes [35], snakes [36], and spiders [37]. Among these enzymes, matrix metalloproteinases (MMPs, also known as matrixins)—a specialized Zn^2+^-dependent subgroup [38]—stand out as particularly important toxic components in jellyfish venom [39]. Proteomic analyses have demonstrated that matrix metalloproteinase-14 and astacin-like metalloprotease toxin 3 precursor form the predominant protein components in *Nemopilema nomurai* venom [39]. Similarly, matrix metalloproteinases comprise approximately 15% of the total protein content in *Stomolophus meleagris* venom [40]. Comparative studies have identified seven and nine distinct MMPs in *Rhopilema esculentum* and *Sanderia malayensis*, respectively [41]. Furthermore, metalloprotease activity has been confirmed in the key lethal toxins of *Cyanea nozakii* [42].

MMPs represent the primary pathogenic mediators in jellyfish envenomation, capable of inducing diverse pathophysiological processes, including severe cardiotoxicity. Notably, Prakash et al. [43] isolated a cardiotoxic component (NnTP) from *N. nomurai* venom (NnV) using chromatographic techniques, subsequently identifying its toxic protease activity. Although direct evidence in jellyfish envenomation remains limited, metalloproteinases are known to exert significant effects on the extracellular matrix, thereby potentially impairing tissue repair processes such as muscle regeneration. For instance, studies on snake venom metalloproteinases—such as those isolated from *Bothrops asper*—have demonstrated that intramuscular injection leads to dysregulated regeneration accompanied by fibrosis and atrophic fibers, suggesting a conserved pathomechanism that may also contribute to the sustained tissue damage observed in severe jellyfish stings [44]. Beyond that, MMPs have been strongly linked to multiple pathological manifestations such as cutaneous damage, inflammatory responses, and secondary infections. Yu et al. further demonstrated that venom metalloproteases critically regulate both pro-inflammatory responses and anti-inflammatory modulation in jellyfish sting-induced dermatitis [45], with pro-inflammatory factor expression primarily controlled through the PI3K-Akt pathway, while anti-inflammatory feedback appears to be mediated via the NF-κB p65 signaling pathway.

### 3.2. Phospholipase A_2_, PLA_2_

Phospholipase A_2_ (PLA_2_) represents another crucial enzymatic component in jellyfish venom, with widespread distribution across various jellyfish species. Research on jellyfish venom enzymes has predominantly investigated PLA_2_ activities, with demonstrated presence in nematocysts from both fishing and mesenteric tentacles of *Cyanea capillata* and *Cyanea lamarckii* [46]. Similar PLA_2_ activity has been confirmed in *C. nozakii* nematocyst venom [47], consistent with the identification of phospholipase motifs in its key lethal toxin (Letoxcn) alongside metalloproteases [42]. Compositional analyses reveal PLA_2_ represents over 20% of the venom components in *S. meleagris* [40]. Further characterization by Heo et al. [48] identified two PLA_2_ subtypes in *N. nomurai* venom: acidic PLA_2_ PA_4_ and PLA_2_ PA_3_A/PA_3_B/PA_5_ variants.

Phospholipase A_2_ (PLA_2_) mediates the hydrolysis of glycerophospholipids at the sn-2 position, releasing lysophospholipids and free fatty acids, including arachidonic acid [49]. This enzyme exhibits particularly high activity in cnidarian tentacles and appendages, where it facilitates critical biological functions such as prey capture, food digestion, and defensive mechanisms. Following cnidarian envenomation, PLA_2_ has been directly linked to diverse pathological effects encompassing pain induction, inflammatory responses, myotoxicity, neurotoxicity, and cytolytic damage [50]. Research demonstrates that PLA_2_ induces hemolysis through two distinct pathways [51,52]—direct enzymatic degradation of erythrocyte membranes and indirect membrane disruption mediated by lytic lipid byproducts generated from phospholipid hydrolysis. The released arachidonic acid and related hydrolysis products further promote the biosynthesis of inflammatory lipid mediators like prostaglandins and leukotrienes, which subsequently propagate inflammatory cascades through their enzymatic activities. These toxic effects may arise either from PLA_2_’s direct action or through synergistic interactions with other venom protein components [53].

### 3.3. Serine Protease

Serine proteases, well-characterized across diverse venomous species including snakes, scorpions, and spiders, primarily manifest hematotoxic effects through coagulation cascade disruption [54]. Recent integrative transcriptomic and proteomic studies have identified these enzymes as additional toxic components in jellyfish venom [40]. Supporting this finding, enzymatic analyses of *N. nomurai* nematocyst extracts detected chymotrypsin-like amidolytic activity that was markedly suppressed by serine protease inhibitors, confirming the presence of chymotrypsin-like serine proteases in jellyfish venom. cDNA sequence analysis revealed that this enzyme shares 41% amino acid sequence identity with human chymotrypsin-like proteins (CTRL and CTRL-1) [55]. Further investigations by Seong Kyeong Bae et al. demonstrated the fibrinolytic capacity of this chymotrypsin-like serine protease, highlighting its potential therapeutic value for thrombotic conditions [56]. Although this protease may participate in jellyfish envenomation-induced inflammatory responses, this potential association awaits further experimental confirmation.

### 3.4. Pore-Forming Toxins, PFTs

Jellyfish pore-forming toxins (PFTs) represent a class of cytolytic proteins capable of directly perforating cellular membranes, exhibiting significant toxicity against target cells and organisms [57]. Initially characterized from lethal box jellyfish nematocysts, including *Carybdea rastonii* [58], *Carybdea alata* [59], *Chiropsalmus quadrigatus* [60], and *C. fleckeri* [61], these toxins demonstrate potent cytotoxicity and lethality across phylogenetically diverse species such as mice, lobsters, and humans [62]. While originally believed to be unique to cubozoans, subsequent research has identified PFTs in other cnidarian classes, including scyphozoans [61] (e.g., *Aurelia* spp. [63], *Cassiopea xamachana* [64]), and hydrozoans (e.g., *Hydra vulgaris* [65]).

Mechanistically, jellyfish PFTs display marked hemolytic and cardiotoxic activities through specific interactions with cellular membranes. These interactions fundamentally compromise membrane integrity and permeability via the formation of transmembrane pores, leading to disruption of ionic gradients. The consequent osmotic imbalance induces cellular swelling and ultimately results in lytic cell death [62].

### 3.5. Others

In addition to the aforementioned proteinaceous and enzymatic components, jellyfish venom contains a diverse array of bioactive toxins with significant pharmacological properties. Notably, potent neurotoxins have been identified that disrupt ion transport dynamics (particularly sodium and calcium fluxes) by inducing channel or pore formation in neuronal and muscle cell membranes [66]. These neurotoxic components can elicit a spectrum of toxicological manifestations, including neurological dysfunction, skeletal myotoxicity, and cardiotoxic effects [67].

**Table 1:** Identified active components of jellyfish venom.

Class	Species	Source	Protein Component	Molecular Weight (kDa)	Bioactivity/Toxicological manifestaion	Major Potential Toxin Families	Ref.
Cardiotoxicity-dominant	*Carukia barnesi*	Double Island (northern Australia)	Crude venom	25–250	pulmonary hypertension; tachycardia	Neural sodium channel activator	[68]
	*Chrysaora quinquecirrha*	Bay of Bengal (India)	Frc-1	105	antioxidant potential	U	[69,70]
			Frc-2	65			
			Frc-3	9			
		Meredith Creek (Maryland)	SNLF	100	cardiotoxic; neurotoxic		
				190			
	*Physalia physalis*	Miami (Florida)	Physalitoxin	240	haemolytic activity	U	[71]
Immunotoxicity-dominant	*Stomolophus meleagris*	Qingdao (China)	SmTX	~45	haemolytic activity	U	[72]
				52			
	*Pelagia noctiluca*	Sicilian coasts (Strait of Messina)	Crude venom	20–100	haemolytic activity	U	[73]
Dual-mechanism synergistic toxicity	*Chironex fleckeri*	Cairns, Townsville, and Weipa (Queensland, Australia)	CfTX-1	43	pore-forming toxins; haemolytic activity	U	[61,74]
			CfTX-2	45			
		Weipa (Queensland, Australia)	CfTX-A	~40			
			CfTX-B	~42			
	*Nemopilema nomurai*	Yellow Sea (South Korea)	Crude venom	15–250	cardiotoxic, hepatotoxic, hemolytic and cytotoxic biological activities	Metalloproteinase, PLA2 activity	[75]
		ROK Coast	JVMP17-1	~25	dermotoxicity, cytotoxicity, and lethality	Metalloproteinase	
			JVMP17-2	50–70			
	*Cyanea capillata*	Isle of Lewis (Western Isles, Scotland)	CcTX-1	31.17	haemolytic activity	Haemolytic proteins	[76,77]
		Isle of Lewis, Rousay (Scotland)	CcNT	8.22	neurotoxin	Neurotoxic peptide	
Unkown	*Cyanea nozakii*	Qingdao (China)	Letoxcn	~50	metalloproteinase activity	U	[42]

This table summarizes toxic components isolated from jellyfish venoms, detailing their molecular characteristics, biological activities, major potential toxin families, and so on. U: Unknown.

## 4. Cardiotoxicity-Dominant Jellyfish Species

Jellyfish venom-induced cardiotoxicity remains one of the most clinically significant and mechanistically complex challenges in marine toxinology. Among various jellyfish species, *Carukia barnesi* and *Chrysaora quinquecirrha* have attracted particular research attention due to their pronounced cardiotoxic effects. Their venoms contain a cocktail of neurotoxins and pore-forming proteins that disrupt cardiac ion channel function, leading to profound disturbances in myocardial electrophysiology.

### 4.1. Carukia barnesi

*Carukia barnesi* (class Cubozoa), commonly known as the Irukandji jellyfish, is a small (~30 mm bell diameter), translucent species [75] predominantly distributed in marine waters from Australia to East Asia [78]. Its venom can induce Irukandji syndrome—a potentially fatal envenomation characterized by sequential pathophysiological phases: initial autonomic storm (profuse diaphoresis and severe anxiety), followed by neuromuscular hyperactivity (painful cramps), and culminating in cardiovascular collapse (acute hypertension progressing to hypotension with cardiac decompensation) [79]. Clinically, approximately 2% of the cases evolve to congestive heart failure within 12–24 h post-sting. Most remarkably, a subset of severe presentations develops Takotsubo cardiomyopathy (TTC) [80], representing the most severe cardiovascular complication of catecholamine-driven toxicosis.

Biochemical analyses have revealed that *C. barnesi* venom contains at least 48 protein components, most with molecular weights below 100 kDa [81]. Comparative proteomic studies further indicate significant differences in protein composition between mature and immature nematocyst venoms. In vitro studies using isolated rat right atria have demonstrated that crude venom extracts from *C. barnesi* elicit a concentration-dependent tachycardic response. This effect was completely abolished by tetrodotoxin (TTX) pretreatment, providing compelling evidence for the presence of sodium channel-activating neurotoxins. Importantly, current evidence suggests that the cardiotoxic effects are mediated primarily through indirect mechanisms involving substantial catecholamine release rather than direct cardiomyocyte injury [68].

### 4.2. Chrysaora quinquecirrha

*Chrysaora quinquecirrha* (Atlantic sea nettle; Cnidaria: Scyphozoa: Semaeostomae) exhibits a broad geographical distribution across tropical and subtropical regions of the Atlantic, Indian, and Pacific Oceans, with particularly high abundance along the eastern coast of the United States. The venom of *C. quinquecirrha* demonstrates marked multi-system toxicity [82], with cardiotoxicity being the most prominent manifestation. Two toxic components (100 kDa and 190 kDa) with potent cardiotoxic and neurotoxic activities have been identified in the tentacle venom of *C. quinquecirrha*, showing a murine median lethal dose (LD_50_) of 0.37 μg/g [70].

Moreover, Kelman et al. isolated toxic proteins from the mesenteric tentacle nematocysts of *C. quinquecirrha*, which were found to significantly suppress spontaneous contractions in cultured chick cardiomyocytes [83]. The venom also induces irreversible contraction in isolated rat aortic rings—an effect that is effectively antagonized by the calcium channel blocker verapamil [84]. These findings collectively indicate that the cardiotoxic mechanism of *C. quinquecirrha* venom involves modulation of voltage-dependent Ca^2+^ channels—a mechanism distinct from the tetrodotoxin-sensitive, catecholamine-mediated cardiotoxicity characteristic of *C. barnesi* envenomation.

### 4.3. Physalia physalis

The siphonophore *Physalia physalis* (Portuguese man o’ war) ranks among the most toxic and globally distributed cnidarians. Mature specimens possess tentacles extending up to 30 m that are densely packed with venomous nematocysts [85], capable of inducing vertebrate paralysis and lethal envenomation in humans. Its venom exhibits both cardiotoxic and hemolytic effects [86], with the hemolytic activity primarily mediated by physalitoxin—a 240 kDa glycoprotein composed of three distinct subunits [71].

Unlike *C. quinquecirrha*, whose calcium-mediated cardiotoxicity is blocked by verapamil, and *C. barnesi*, whose TTX-sensitive toxicity is catecholamine-driven, its cytotoxic mechanism involves direct membrane permeabilization, inducing pathological calcium influx and dose-dependent damage in embryonic chick cardiomyocytes [87,88]. The venom also demonstrates potent neurotoxicity, causing dose- and time-dependent locomotor deficits in Drosophila that implicate bioactive components targeting central nervous system ion channels [89].

## 5. Immunotoxicity-Dominant Jellyfish Species

Recent studies demonstrate that jellyfish envenomation can trigger both immediate and delayed hypersensitivity reactions, with documented cross-reactivity between different jellyfish venoms. While systemic anaphylaxis following envenomation remains relatively uncommon, repeated exposure among coastal residents and maritime workers substantially increases sensitization risk. Notably, allergic responses may be initiated through either dermal contact or accidental ingestion.

Immunological investigations reveal that specific jellyfish species contain venom components with marked immunomodulatory capacity. For instance, jellyfish-derived collagen extracts and peptides potently stimulate pro-inflammatory cytokine release, particularly tumor necrosis factor-alpha (TNF-α) and interferon-gamma (IFN-γ) [86]. Furthermore, both proteinaceous toxins and carbohydrate-rich components (e.g., glycoproteins and polysaccharides) from nematocysts can function as antigens, inducing clinical symptoms through cellular and/or humoral immune pathways [27]. The subsequent discussion will examine immunodominant jellyfish species, detailing their distinct clinical presentations and the molecular mechanisms underlying their pathogenic effects.

### 5.1. Stomolophus meleagris

The rhizostome jellyfish *Stomolophus meleagris* (Cnidaria: Scyphozoa) is a large species (bell diameter: 180–980 mm) widely distributed across China’s coastal waters from the Yellow Sea to the South China Sea. Its venom contains the characteristic hemolytic protein SmTX, purified through sequential anion-exchange and gel-filtration chromatography. This multi-subunit toxin, composed of ~45 kDa and 52 kDa subunits, exhibits potent hemolytic activity with optimal function at pH 5.0 and temperatures between 23 and 45 °C [72].

Experimental studies confirm that *S. meleagris* venom triggers significant acute inflammatory responses. In murine footpad injection models, the venom induced dose-dependent edema and erythema. In vitro analyses of TE-treated HaCaT cells revealed substantial upregulation of pro-inflammatory cytokines (IL-1β, IL-6, IL-8, and TNF-α), while Western blotting demonstrated concurrent activation of both NF-κB and MAPK signaling pathways [90].

### 5.2. Pelagia noctiluca

*Pelagia noctiluca* (Cnidaria: Scyphozoa), a bioluminescent jellyfish species, represents one of the most medically significant medium-sized cnidarians in Mediterranean waters [28]. Its venom elicits both localized pathologies (erythema or edema) and systemic inflammatory responses following envenomation. Frazão et al. [91] revealed 68 proteins in *P. noctiluca* venom by proteomics, including zinc metalloproteinases with the ShK toxin structural domain, and the molecular weight distribution of haemolytically active proteins in the venom was in the range of 20–100 kDa [73].

Accidental contact with *P. noctiluca* often induces severe localized and systemic pathologies [92], primarily mediated by the venom’s potent pro-inflammatory properties [93]. Experimental investigations in rat models demonstrate that venom exposure leads to pronounced inflammatory cell infiltration (predominantly neutrophils and macrophages) in pulmonary and intestinal tissues within 6 h post-injection [94]. This cellular response correlates with upregulated NF-κB p65 expression. Interestingly, pretreatment with the antioxidant tempol significantly attenuates these pathological changes, confirming the essential role of ROS-mediated NF-κB pathway activation in the inflammatory cascade.

## 6. Dual-Mechanism Synergistic Toxicity

Jellyfish venoms demonstrate significant interspecies variability in their bioactive components, with cardiotoxic and immunomodulatory effects traditionally studied as distinct phenomena. However, recent studies have identified species (e.g., *C. fleckeri*) whose venoms simultaneously target both cardiac and immune systems—where direct myocardial toxicity and systemic inflammatory responses act synergistically to amplify tissue damage. This section systematically examines jellyfish species with dual cardiotoxic-immunomodulatory venom actions, characterizing their toxic constituents and delineating the mechanistic basis of their combined pathological effects.

### 6.1. Chironex fleckeri

*Chironex fleckeri* (commonly known as the Australian box jellyfish or sea wasp), predominantly found in northern Australian coastal waters, is the most toxic cubozoan jellyfish known. Its venom can cause severe dermatonecrosis, systemic inflammatory responses, and life-threatening cardiotoxicity in humans. The venom contains two major classes of pore-forming toxins: CfTX-1/2 (43–45 kDa) and CfTX-A/B (~40–42 kDa), both belonging to the porin family but phylogenetically distinct [61]. Among these, CfTX-1 and CfTX-2 are identified as type I cardiotoxins and represent the key mediators of acute cardiovascular failure. They induce rapid cardiac arrest in mice, even at ultralow doses, via direct action on cardiomyocytes [95]. Structural studies attribute CfTX-1’s potent activity partly to its unique helical domain [96]. In contrast, CfTX-A and CfTX-B exhibit minimal cardiotoxicity but demonstrate strong hemolytic activity [74].

Both toxin classes are highly immunogenic and capable of eliciting specific antibody responses. Recombinant CfTX-1 shows particularly robust immunoreactivity, supporting its potential as a vaccine candidate [97]. To date, CfTX-1 and CfTX-2 remain unique to *C. fleckeri* and are considered the principal toxins responsible for the rapid cardiovascular collapse following envenomation. The venom thus exerts synergistic cardiotoxic and immunological effects through the coordinated action of its constituent porins.

### 6.2. Nemopilema nomurai

*Nemopilema nomurai* is recognized as one of the most dangerous large jellyfish species inhabiting East Asian coastal waters, where it is responsible for an estimated 100,000 envenomation cases annually. Proteomic analysis has revealed that its venom contains a complex mixture of over 150 distinct protein components [75], including key toxins such as metalloproteases and phospholipases. Of particular significance, researchers have isolated 13 toxin homologs from the lethal fraction of *N. nomurai* specimens obtained from Dalian, China, comprising phospholipases, potassium channel inhibitors, and hemolysins. Among these, alkaline phospholipases have been shown to play a critical role in mediating the venom’s lethal effects [98].

Most *N. nomurai* envenomations primarily cause localized cutaneous symptoms, including acute pain and inflammatory responses. In vitro studies reveal that venom exposure triggers dose-dependent increases in both mRNA and protein expression of key pro-inflammatory mediators (TNF-α, IL-6, and MCP-1) in skin cell lines (HaCaT and CCC-ESF-1) [99]. Beyond its dermatological effects, the venom also directly suppresses cardiac function through negative inotropic and chronotropic actions [100]. This cardiotoxicity is particularly selective, as evidenced by the venom’s pronounced cytotoxicity toward cardiomyocytes (H9c2 cell line), which alters the expression of 72 cardiac-related proteins—including established heart failure biomarkers like annexin A2 and myosin-7 [101]. Notably, this cardiac toxicity far exceeds the venom’s impact on skeletal muscle cells (C2C12) [102], highlighting its specific targeting of myocardial tissue.

### 6.3. Cyanea capillata

The lion’s mane jellyfish (*Cyanea capillata*), a dominant large jellyfish species in southeastern Chinese coastal waters with bell diameters often exceeding 1 m, produces venom exhibiting distinct cardio-immunological synergistic toxicity. The hemolytically active protein CcTX-1 was isolated from the tentacle venom of *C. capillata*. This toxin exists in two isoforms, with the predominant subtype exhibiting a molecular mass of 31.17 kDa. Notably, sequence alignment demonstrates high homology between CcTX-1 and known cubozoan hemolysins [76], suggesting potential functional similarities. In addition to its hemolytic components, *C. capillata* venom contains the low-molecular-weight neurotoxin CcNT (8217.4 Da), which induces paralysis via selective inhibition of voltage-gated sodium channels [77]. Together, these findings highlight the multifunctional nature of *C. capillata* venom, combining both hemolytic and neurotoxic mechanisms to exert its potent biological effects.

The lion’s mane jellyfish (*Cyanea capillata*), a prevalent species in southeastern Chinese coastal waters, produces venom with cardio-immunological synergistic toxicity. Its tentacle venom contains two key toxins: the hemolytically active protein CcTX-1 (31.17 kDa main isoform), which shows high homology to cubozoan hemolysins [76], and the low-molecular-weight neurotoxin CcNT (8.2 kDa), which inhibits voltage-gated sodium channels and induces paralysis [77]. These components act synergistically, enabling the venom to exert combined hemolytic and neurotoxic effects.

**Figure 3:**
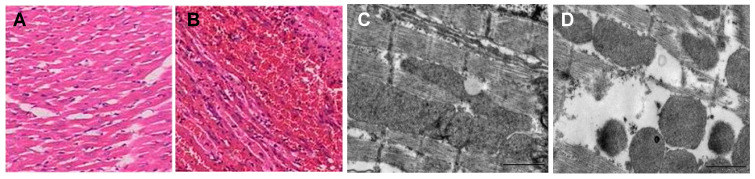
Pathological and ultrastructural changes in rat heart following TOE administration (5 mg/kg i.v., 60 min). (**A**,**B**) Hematoxylin-eosin (HE) staining of cardiac tissue (400× magnification): (**A**) Normal cardiac tissue architecture in control group. (**B**) Severe hemorrhage observed in cardiac tissue after TOE administration. (**C**,**D**) Transmission electron microscopy (TEM) of rat cardiomyocytes (scale bars: 1 μm): (**C**) Normal ultrastructure of control cardiomyocyte, showing intact sarcomeres with distinct Z-lines, and mitochondria with well-defined cristae. (**D**) TOE-treated group exhibiting disrupted and dissolved sarcomeric filaments and partial mitochondrial swelling, while mitochondrial cristae remained visible [104].

Experimental studies demonstrate that *C. capillata* venom induces significant inflammatory responses. Salmon exposure experiments revealed acute tissue damage, including edema and epithelial detachment, accompanied by sustained inflammatory reactions [103]. Regarding cardiotoxicity, tentacle extract (TOE) from *C. capillata* exhibits direct cardiac toxicity in rat models (Figure 3) [104]. Intravenous administration caused dose-dependent myocardial injury, culminating in acute global heart failure. In vitro heart perfusion assays confirmed these findings, showing rapid reductions in both heart rate (HR) and left ventricular developed pressure (LVDP) following TOE exposure. Notably, administration of 240 μg TOE triggered severe arrhythmias and cardiac arrest. Histopathological examination identified characteristic myocardial lesions, including wavy fiber distortion. Mechanistic studies suggest this toxicity may involve overactivation of L-type Ca^2+^ channels and adrenergic receptors [105].

## 7. Treatment Strategy

In summary, systematic analysis of jellyfish venom toxicity reveals that cardiotoxic and immunomodulatory effects are not mutually exclusive pathological manifestations. While one may dominate clinically, these effects frequently coexist and interact synergistically in envenomed patients. Targeting the bioactive components responsible for these pathological effects may hold significant therapeutic potential. Given the substantial interspecies variations in venom composition and pathogenic mechanisms among jellyfish, it is imperative to establish toxin profile-based personalized treatment protocols. Therapeutic strategies should be tailored according to the envenomation type and clinical manifestations, with precise identification of the jellyfish species and timely intervention being critical determinants of treatment efficacy. For cardiotoxicity-dominant jellyfish envenomations, clinical management should prioritize stabilization of myocardial electrophysiological activity, supplemented by inflammatory storm control during the subacute phase. Conversely, for immunodominant jellyfish envenomations, targeted immunotherapy against immune-mediated symptoms may yield superior clinical outcomes. And for cardio-immunological synergistic envenomations, a dual-blockade strategy combining toxin neutralization (e.g., antivenom) with immunomodulatory intervention is essential to address both pathological pathways concurrently. This section synthesizes targeted antagonists and therapeutic strategies corresponding to distinct toxicological mechanisms, aiming to establish an evidence-based framework for clinical management of jellyfish envenomations.

### 7.1. Comprehensive Treatment of Jellyfish Sting

Jellyfish venom exhibits complex composition with inherent instability, where the severity of local and systemic reactions depends on multiple variables: the specific venom profile, delivered toxin quantity, dermal contact area with tentacles, exposure duration, and so on. For jellyfish envenomation, basic first-aid measures can be categorized into three primary objectives: (i) preventing further nematocyst discharge, (ii) mitigating local venom effects (pain and tissue damage), and (iii) controlling systemic reactions.

Prompt removal of residual tentacles and nematocysts from the skin is critical post-envenomation, as retained tentacular fragments can continue discharging venom, leading to secondary envenomation events [106]. Significant controversy persists regarding optimal tentacle removal techniques for minimizing local envenomation symptoms, particularly concerning the use of oral/topical analgesics, hot water, ice packs, or vinegar. This debate stems primarily from interspecies variations in venom composition and pathological effects.

Morabito et al. recommend immediate application of lidocaine, acetic acid, ethanol, or ammonia solutions to *Pelagia noctiluca* sting sites, demonstrating significant pain relief and adverse reaction reduction [107]. In contrast, Burnett et al. found that topical lidocaine monotherapy failed to provide adequate analgesic efficacy in their clinical studies [108].

Hot water immersion or heat packs remain widely used for marine envenomation management, yet considerable disagreement persists among researchers regarding the comparative efficacy of thermotherapy versus cryotherapy. Cryotherapy using ice packs or cold compresses has been demonstrated to effectively limit both inflammatory progression and venom spread, while concurrently providing analgesic benefits [109]. For scyphozoan envenomations (e.g., *Aurelia aurita*, *C. fleckeri*, or *S. meleagris*), thermotherapy appears to confer greater clinical benefit [106]. This phenomenon may stem from the thermolabile nature of these jellyfish venoms and/or direct modulation of nociceptor activity by heat immersion, collectively contributing to observed pain reduction [105]. In contrast, for *C. quinquecirrha*—a species with relatively thermostable venom [110]—both thermotherapy and cryotherapy demonstrate limited clinical efficacy [111].

Given the inherent accessibility of seawater, victims often instinctively employ it for initial rinsing. While this approach has demonstrated efficacy in tentacle removal, pain relief, and nematocyst inhibition [112], Doyle et al. found that seawater irrigation after *C. capillata* envenomation exacerbates stinging pain and significantly enhances venom dispersal—effects not observed with vinegar application [112]. Conventional remedies including urine [113], ammonia, 70% ethanol, and isopropanol demonstrate limited efficacy for most jellyfish envenomations, with potential to paradoxically stimulate nematocyst discharge [10].

In summary, no universal consensus exists regarding optimal first-aid protocols for jellyfish stings, primarily due to substantial interspecies variability in venom composition and pathophysiological effects. We recommend immediate implementation of species-specific treatment protocols when the envenoming jellyfish is definitively identified. For unclassified envenomations, a comprehensive risk–benefit assessment integrating all the relevant factors—including individual physiological variability and envenomation severity—is imperative for treatment decision-making.

### 7.2. Therapeutic Strategies for Cardiotoxicity

**Figure 4:**
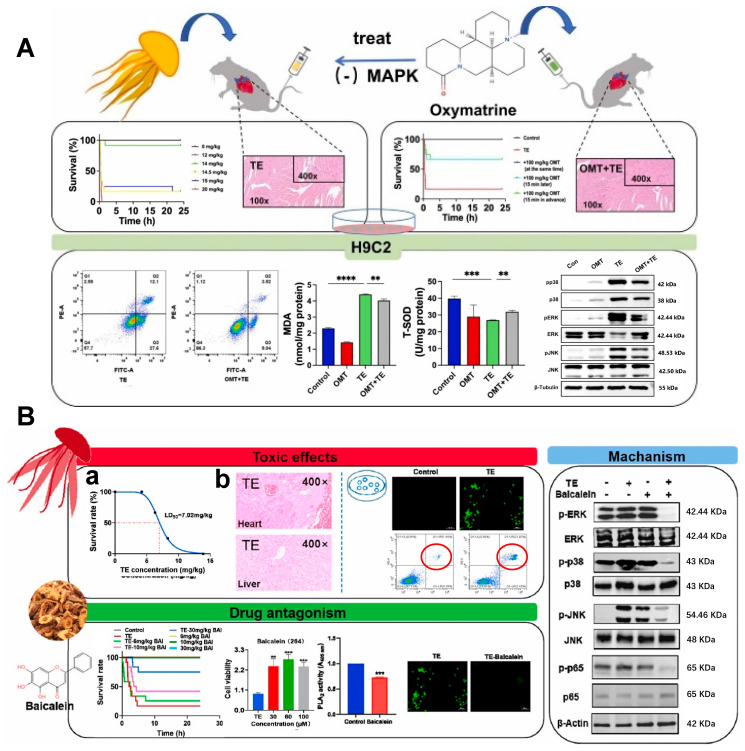
(**A**) Oxymatrine antagonizes oxidative stress and apoptosis in *N.nomurai* toxin-induced cardiotoxicity by inhibiting mitogen-activated protein kinase. ** *p* < 0.01, *** *p* < 0.001, **** *p* < 0.001, ns means no significance [115]. (**B**) Baicalein reduces mortality in mice and alleviates cardiomyocyte injury induced by *R.esculentum* toxin through regulation of the ROS/MAPK/NF-κB pathway. (**a**) LD_50_ curve of TE (*n* = 12). (**b**) Pathological section examination (*n* = 3). The scale bar in the reactive oxygen spices fluorescence imaging represents 100 μm. ** *p* < 0.01, *** *p* < 0.001, ns means no significance [116].

For cardiotoxicity-dominant jellyfish envenomation, therapeutic strategies should prioritize neutralizing cardiac-specific pathological pathways. The resulting cardiotoxicity typically involves interconnected mechanisms, including rhythm disturbances resulting from altered cardiomyocyte membrane permeability (Figure 4). Central to this process is the disruption of myocardial calcium homeostasis—a well-established mechanism underlying venom-induced cardiac dysfunction [114]—and emerging evidence also implicates cytokine dysregulation in this pathological cascade [101].

As already established, calcium signaling plays a central role in cardiac excitation–contraction coupling, and its dysregulation can lead to severe consequences such as arrhythmias, cardiomyocyte injury, and programmed cell death [117]. Accordingly, calcium channel blockers have shown significant cardioprotective effects against jellyfish venom: felodipine mitigates cardiac damage caused by *N. nomurai* venom [100], while nifedipine and verapamil improve impaired cardiac function following *C. capillata* tentacle extract (TOE) exposure [105]. Current pharmacological interventions, thus, predominantly target these pathways using calcium channel blockers and antivenom therapies.

Antivenom (AV) therapy remains the primary intervention for *C.fleckeri* envenomation, with the Commonwealth Serum Laboratories (CSL) antivenom being the only commercially available treatment for cubozoan stings worldwide. While prophylactic antivenom administration has demonstrated survival benefits in murine models [118] and reduced cardiovascular collapse induced by *C. fleckeri* venom [119], conflicting data exist regarding its therapeutic window. Notably, AV given 15 min pre-envenomation failed to significantly alleviate toxicity in animal studies [120], leaving current evidence on efficacy and optimal dosing inconclusive. Interestingly, when the venom was incubated with either AV for 3 h prior to infusion, the effect of the venom was almost abolished. This result suggests that the venom acts extremely rapidly, and the clinical efficacy of AV is limited by the difficulty of administering it sufficiently early after envenomation. However, in vitro studies by Andreosso et al. demonstrated that antivenom at 5-fold the maximum recommended clinical dose significantly improved cell viability, with this protection showing dose-dependent enhancement at higher concentrations [121]. Considering venom-induced cardiotoxicity, one study explored combination therapy using antivenom with verapamil, demonstrating enhanced efficacy and prolonged survival in envenomed mice [122]. It should be noted that this approach remains contentious, as verapamil’s benefits decrease with escalating antivenom doses, and the ideal therapeutic ratio requires further clarification. Additionally, the extraction techniques used to obtain venom for AV production may result in antiserum lacking critical antibodies, thereby contributing to inconsistent and contradictory experimental outcomes.

Oxidative stress plays a critical role in various cardiac pathological processes and contributes to numerous cardiovascular abnormalities. Accumulating evidence suggests that the cardiotoxicity induced by jellyfish envenomation may be associated with inflammatory responses and oxidative damage [15,18]. Therefore, targeting MAPK activation and oxidative stress represents a promising therapeutic strategy for mitigating jellyfish venom-induced cardiotoxicity. Dong et al. revealed that oxymatrine (OMT) effectively counteracted the lethal effects induced by tentacle extract (TE) of Nemopilema nomurai, reducing cardiac edema and myocardial fiber disruption. In vitro experiments further demonstrated that OMT attenuated TE-induced decreases in H9C2 cell viability and inhibited apoptosis [115]. Similarly, Wang et al. reported that pretreatment with baicalein significantly improved survival rates in mice exposed to tentacle extract from Rhopilema esculentum. Baicalein was shown to alleviate toxin-induced oxidative stress and cell death by modulating the ROS/MAPK/NF-κB pathway, concurrently ameliorating TE-triggered cardiac injury [116].

Moreover, cardiac glycogen synthesis capacity plays an essential role in normal heart development. Notably, as a central regulatory pathway in cardiac failure (CF) [123], glycogen metabolism demonstrates therapeutic potential for jellyfish venom-induced CF through maintaining storage capacity and enhancing energy production efficiency. Qin et al. [124] identified via single-cell transcriptome analysis that the energy-regulating protein α-1 acid glycoprotein (AAG), a key modulator of glycogen metabolism, was significantly upregulated in CF patients following jellyfish envenomation. These findings were further validated in animal studies using a toxin-induced CF mouse model, where AAG deficiency accelerated heart failure progression, while either exogenous supplementation or endogenous upregulation of AAG effectively reversed cardiac dysfunction. This discovery provides a novel targeted therapeutic strategy for jellyfish venom-associated CF.

### 7.3. Therapeutic Strategies for Immunotoxicity

The clinical management of immunodominant jellyfish envenomations poses distinct therapeutic challenges compared to cardiotoxicity-dominant cases. Nematocyst-derived components—such as chitin, collagen, and polysaccharides—can chronically stimulate innate immune cells (e.g., keratinocytes, dendritic cells, and mast cells) [27], leading to persistent vesiculobullous and pruritic skin reactions. In severe cases, such as envenomation by *N. nomurai*, the venom can trigger a deadly cytokine storm—a systemic and dysregulated inflammatory response that significantly contributes to multi-organ failure and mortality [18]. However, current research on immune-mediated jellyfish envenomation predominantly centers on cutaneous pathologies, especially jellyfish dermatitis.

As previously discussed, matrix metalloproteinases (MMPs) are pivotal mediators of both cutaneous damage and inflammatory responses following jellyfish envenomation. Mechanistic investigations indicate that venom-derived MMPs primarily exert their pathogenic effects by activating MAPK and NF-κB signaling pathways, which are known to be key regulators in the development of jellyfish dermatitis. Owing to this central role, MMP inhibitors have gained attention as potential therapeutics. Li et al. [122] recently demonstrated that batimastat and disodium EDTA, as MMP inhibitors, show marked efficacy against *N. nomurai* venom (NnNV)-induced dermatitis.

**Figure 5:**
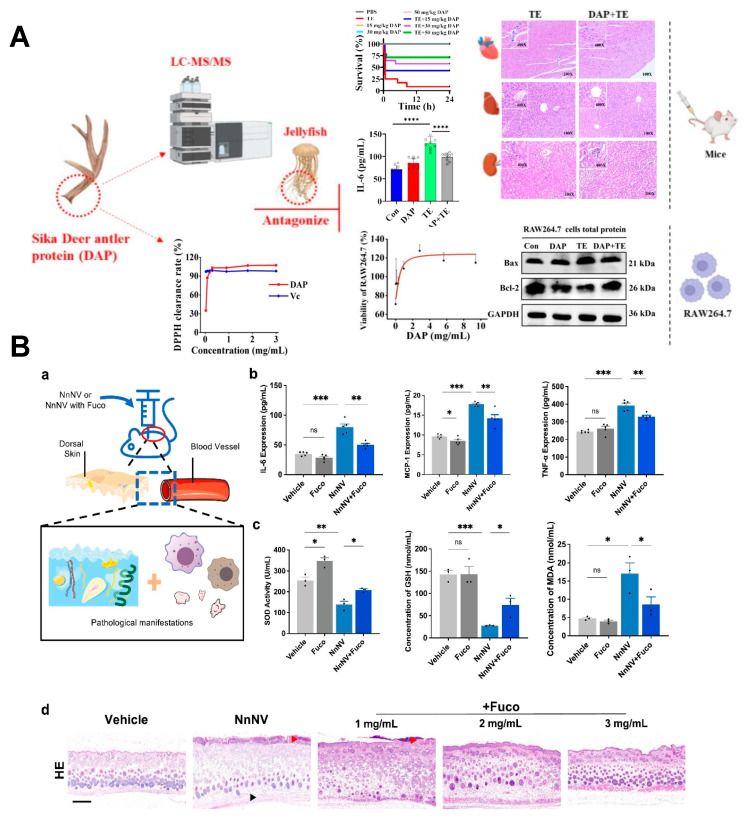
(**A**) Sika deer antler protein antagonizes the inflammatory response and oxidative damage induced by jellyfish venom [126]. **** *p* < 0.001. (**B**) Fucoidan alleviated NnNV-induced skin inflammatory stimulation effect in mice. (**a**) Graphical depiction of the inflammatory effects of NnNV. (**b**,**c**) Protein expression levels of inflammatory factors (IL-6, TNF-α, and MCP-1) and oxidative stress indicators in mouse blood (3 mg/mL). * *p* < 0.05, ** *p* < 0.01, *** *p* < 0.001, ns means no significance. (**d**) Pathological section of dermal toxicity. The scale bars of histopathological section are 100 μm. Red arrowheads indicate deepening of epidermal staining. Black arrowheads indicate damaged subcutaneous muscle tissue [16].

Oxidative stress may act synergistically with inflammatory responses [125], as intracellular ROS accumulation activates signaling pathways including MAPK, which subsequently exacerbates inflammatory cascades. Therefore, therapeutic interventions could target not only direct MMP inhibition but also MAPK/NF-κB signaling blockade and oxidative stress reduction (Figure 5). In particular, directly targeting the NF-κB signaling pathway has been shown to mitigate harmful effects of jellyfish envenomation. Xiao et al. have demonstrated that dexamethasone, a known inhibitor of the NF-κB pathway, effectively suppresses cytokine storm and mitigates organ damage, thereby improving survival rates in a mouse model of delayed jellyfish envenomation syndrome [18]. Similarly, Liu et al. [90] showed that troxerutin effectively mitigates *S. meleagris* venom-induced cutaneous toxicity and inflammation through dual suppression of MAPK and NF-κB pathways. Compounds such as fucoidan [16] and deer antler protein (DAP) [126] have been demonstrated to decrease key inflammatory markers (IL-6, IL-1β, and TNF-α) in murine models by inhibiting these same pathways, ultimately enhancing survival rates after *N. nomurai* venom exposure.

While acute cutaneous reactions are common following jellyfish envenomation, certain cases exhibit delayed and recurrent dermatological manifestations [127]. For such persistent symptoms, immunomodulators—including pimecrolimus and tacrolimus—have shown therapeutic efficacy by specifically inhibiting T-lymphocyte activity.

Collectively, these findings underscore that anti-inflammatory drugs represent a promising strategy for treating jellyfish envenomation, warranting further clinical investigation. Future research should focus on identifying additional inflammatory signaling pathways and immune cell interactions to uncover novel therapeutic targets. This approach reflects a strategic shift from merely neutralizing venom toxins toward actively modulating the host immune response—a potentially more practical and universally applicable paradigm given the vast interspecies diversity of jellyfish and their venom compositions.

## 8. Summary and Outlook

Jellyfish envenomations constitute the most common marine biological injuries and have become a major public health issue worldwide. However, the pathogenic mechanisms and clinical treatment strategies for jellyfish envenomation remain insufficiently elucidated. Current therapeutic approaches frequently address individual symptoms rather than the comprehensive, multi-system effects of jellyfish venoms. Here, we propose that species-specific treatment protocols could improve clinical outcomes, as jellyfish venoms have diversified evolutionarily to meet distinct ecological demands—such as the potent cardiotoxins of predatory species like *C. fleckeri* for rapid immobilization, or the immunotoxins used by competition-inhibiting species like *S. meleagris* to elicit inflammatory deterrence [128]. It is important to note, however, that while tailored treatments show promise, a more pragmatic and impactful long-term strategy emphasizes prevention. Effective public health measures—including bloom monitoring, predictive risk modeling, protective netting, and public education on avoidance and first aid—can substantially reduce exposure and health burdens. Integrating prevention into coastal management and health policy frameworks offers a sustainable approach to mitigating the growing threat of jellyfish envenomations.

Furthermore, it is essential to acknowledge the limitations inherent in current research paradigms. Studies on cardiotoxicity have largely relied on isolated myocardial models, which may overlook systemic effects mediated by venom components. Similarly, investigations of immune responses have predominantly focused on localized cutaneous reactions, leaving the interplay between immune activation and cardiac injury poorly understood. The full pathological sequence—from venom penetration through the skin and hematogenous spread to end-organ damage—likely involves mechanisms far more complex than currently recognized.

While the majority of jellyfish stings are mild and self-limiting, a small yet clinically significant subset leads to severe complications that account for the majority of envenomation-related mortality. The underlying mechanisms—particularly the systemic immune processes driving cardiac injury and dysregulated inflammatory responses—remain poorly elucidated. Future research should employ an integrated multi-omics strategy—combining proteomic, transcriptomic, and metabolomic approaches—to comprehensively characterize key pathogenic venom constituents and elucidate the dynamic spatiotemporal interactions between immune activation and cardiac dysfunction. Translating these mechanistic insights into clinical applications will require a clear translational pathway. This includes the development of physiologically relevant preclinical models (e.g., murine cardiotoxicity models or human cell-based platforms) that accurately recapitulate human immune–cardiac interactions, alongside rigorous biomarker validation for early detection of severe envenomation. Furthermore, overcoming regulatory challenges for venom-based therapies—particularly for rare severe cases—will depend on international collaboration to standardize venom extraction, toxin characterization, and clinical endpoint definitions. Successfully bridging basic science and clinical practice will enable the development of mechanism-driven interventions, such as targeted antivenoms, tailored nanoparticle delivery systems for toxin-specific neutralization, and adjunctive immunomodulatory therapies. These advances may ultimately pave the way for precision medicine approaches in jellyfish envenomation management.

## Data Availability

Not applicable.
